# A paradoxical role for sestrin 2 protein in tumor suppression and tumorigenesis

**DOI:** 10.1186/s12935-021-02317-9

**Published:** 2021-11-16

**Authors:** Junsheng Qu, Moyi Luo, Jingwen Zhang, Fang Han, Ningning Hou, Ruiyan Pan, Xiaodong Sun

**Affiliations:** 1 Department of Endocrinology and Metabolism, Affiliated Hospital of Weifang Medical University, 2428 Yuhe Road, Weifang, 261031 Shandong China; 2 School of Clinical Medicine, Weifang Medical University, Weifang, China; 3 Clinical Research Center, Affiliated Hospital of Weifang Medical University, Weifang, China; 4 Department of Pathology, Affiliated Hospital of Weifang Medical University, Weifang, China; 5 School of Pharmacy, Weifang Medical University, Weifang, China

**Keywords:** Sestrin 2, Cancer, Tumor suppressor, Tumorigenesis

## Abstract

Sestrin 2, a highly conserved stress-induced protein, participates in the pathological processes of metabolic and age-related diseases. This p53-inducible protein also regulates cell growth and metabolism, which is closely related to malignant tumorigenesis. Sestrin 2 was reported to regulate various cellular processes, such as tumor cell proliferation, invasion and metastasis, apoptosis, anoikis resistance, and drug resistance. Although sestrin 2 is associated with colorectal, lung, liver, and other cancers, sestrin 2 expression varies among different types of cancer, and the effects and mechanisms of action of this protein are also different. Sestrin 2 was considered a tumor suppressor gene in most studies, whereas conflicting reports considered sestrin 2 an oncogene. Thus, this review aims to examine the literature regarding sestrin 2 in various cancers, summarize its roles in suppression and tumorigenesis, discuss potential mechanisms in the regulation of cancer, and provide a basis for follow-up research and potential cancer treatment development.

## Introduction

Cancer is a category of malignant diseases where cell proliferation and survival are uncontrolled [1]. Cancer is the second leading cause of death worldwide following cardiovascular disease [2]. Emerging evidence has shown that the molecular mechanisms of cancer progression and suppression are quite complex. Recent studies demonstrated that the processes of tumor development and progression involved transcription factors [3], microRNAs [4, 5], long noncoding RNAs [6], circular RNAs [7], cytokines [8, 9], exosomes [10], inflammasomes [11], immune microenvironment [12], hormones [13], and other potential therapeutic protein targets [14, 15]. Various molecular targeted therapies have been developed to treat malignancies because of their specificity, efficacy, improved patient tolerance, and lower toxicity [16]. Therefore, the exploration and discovery of effective potential molecule targets are important to significantly improve cancer treatment.

**Fig. 1:**
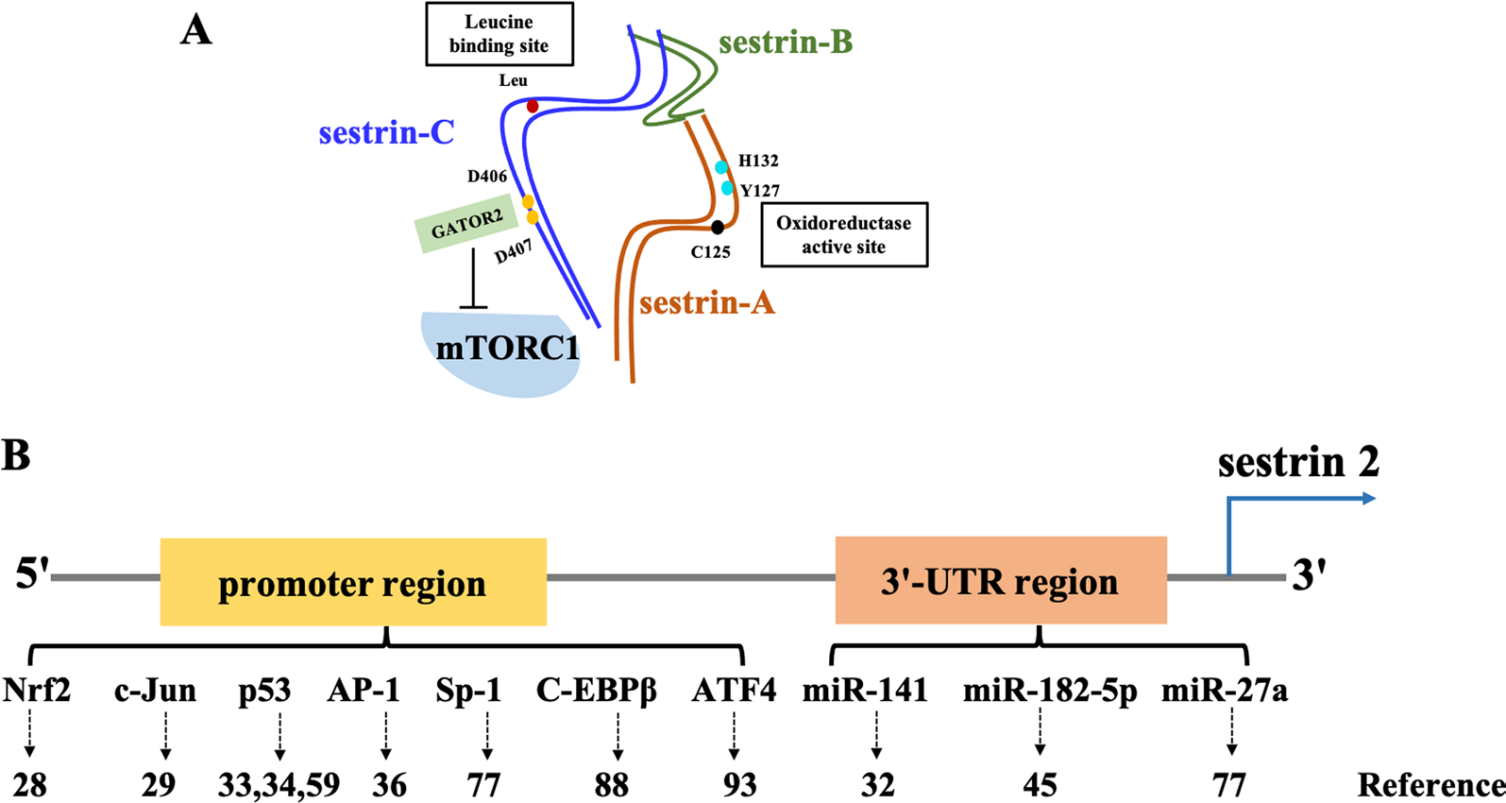
A schematic diagram showing the structure and transcription regulators of sestrin 2. **A** The functional domains of human sestrin 2 (sestrin-A, sestrin-B, and sestrin-C). In sestrin-A, the cysteine residue (C125) and conserved residues of the proton relay system (Y127 and H132) are important for its antioxidant activity. In sestrin-C, the two surface-exposed aspartates (D406 and D407, DD motif) are responsible for interacting with GATOR2 to inhibit mTORC1 signaling. **B** Sestrin2 expression plays a central role in cancer regulated by multiple transcription factors and miRNAs

The sestrin proteins comprise three subtypes: sestrin 1, sestrin 2, and sestrin 3 [14]. Sestrin 2, a homolog of the p53-activated gene 26, is encoded by the hypoxia-inducible gene 95 [17]. Sestrin 2, a highly conserved stress-induced protein, is mainly expressed in mammals and secreted by various immune and non-immune cells, such as macrophages, T lymphocytes, and epithelial cells [18–21]. Human sestrin 2 has three sub-domains (Fig. 1A): sestrin-A, sestrin-B, and sestrin-C [22]. The physiological function of sestrin 2 is mainly dependent on the symmetrical structures of the sestrin-A and sestrin-C domains, which have similar spherical structures but different functions that are regulated by various transcription regulators (Fig. 1B) [23]. The structured domain of sestrin-A is regarded as an alkyl hydroperoxide reductase, which is responsible for antioxidant activity. Sestrin-B is a linker that connects A and C. Sestrin-C is a leucine binding site that acts as a leucine sensor in the mammalian target of rapamycin (mTOR) complex 1 (mTORC1) pathway. The interaction between sestrin-C and the GTPase-activated RAG protein complex is essential for regulating 5ʹ-adenosine monophosphate-activated protein kinase (AMPK) and mTORC1 signaling by sestrin 2 [18, 24]. Under the conditions of DNA damage, oxidative stress, hypoxia and nutritional deficiency, sestrin2 can maintain cell homeostasis by activating AMPK and inhibiting mTOR signaling pathways [14]. Furthermore, sestrin 2 plays roles in maintaining cellular activity, antioxidation, mitochondrial homeostasis and in regulating autophagy [14]. Besides, studies have demonstrated that sestrin 2 is associated with immune system diseases, liver diseases, ischemic-reperfusion lesions, neurodegenerative diseases, cardiovascular disorders, aging, and cancer [14, 18, 25–27].

Recent studies have shown that sestrin 2 is a cancer biomarker and potential therapeutic target that is critical for cancer occurrence and development [14, 18, 28]. Sestrin 2 is involved in many processes, such as tumor cell proliferation; apoptosis; autophagy; anoikis resistance; drug resistance; sensitivity to radiotherapy; tumor differentiation; tumor, node, and metastasis (TNM) staging; lymphatic metastasis; and patient survival [29–32]. Alterations in sestrin 2 expression have been found in a majority of cancers (Table 1). Moreover, sestrin 2 function is different in several cancers. Sestrin 2 was verified as a tumor suppressor gene in some studies, while other reports regarded sestrin 2 as an oncogene. As a novel diagnostic and therapeutic target, the paradoxical role of sestrin 2 in cancers may hinder clinical application in the future. Therefore, it is of great significance to investigate the possible reasons behind these contradictions. In this review, we will examine the state of research on sestrin 2 in different types of cancers and demonstrate its role in inhibition and promotion of cancer by focusing on related mechanisms.

**Table 1:** The expression, biological function, and mechanism of Sestrin2 in cancers

Tumor	Cell type/model	Sestrin2 expression	Biological function of Sestrin2		Mechanism	References
CRC	CRC patients:279	Down	Suppressor gene	Suppresses tumor invasion and metastasis	N/A	[30]
	Human CRC cells (HT-29, SW480, SW620 and LoVo)			Prognostic factor for CRC		
	A mouse xenograft model			Suppresses CRC xenografts growth		
CRC	Oncomine analysis	Down	Suppressor gene	Inhibits colitis and colon carcinogenesis	Inhibited ER stress and mTORC1	[33]
SGC	Eyelid SGC tumour:20	Down	Suppressor gene	Correlate with tumor differentiation and upper eyelid involvement	N/A	[34]
NB	50 NB (GEO ID: GSE12460)	Down	N/A	Correlate with prognosis	Through Sestrin2/mTORC1 pathway	[35]
				Participates in the regulation of autophagy by LSD1		
Bladder cancer	Bladder cancer patients:10	Down	Suppressor gene	Participates in isorhapontigenin-induced autophagy	Via a MAPK8-JUN-Sestrin2-dependent manner	[36]
Prostate cancer	Prostate cancer cells (LNCaP clone FGC, DU145, PC3)	Down	Suppressor gene	Inhibits cell proliferation and increases radiosensitization	N/A	[37]
SCC and Melanoma	SCC, malignant melanoma, and metastatic melanoma samples	Up	Oncogene	Inhibits apoptosis, induces drug resistance	Activates AKT through a PTEN dependent manner	[38]
	Human squamous carcinoma cell (A431), human amelanotic melanoma cells (A375, MEL624)					
	A431, A375/xenograft tumor model					
Metastatic melanoma	Metastatic melanoma tissue microarray: 45	Up	Oncogene	Serve as a predictor of poor prognosis	Reduces intracellular ROS	[39]
	Metastatic melanoma cells (A375, A2058, UACC257,451Lu and HTB67)			Promotes anoikis resistance and distant metastasis		
	A2058/xenograft tumor model					
EC	Endometrial cancer: 6	Up	N/A	Relates to the overall survival and disease-free survival	Dependent on the mTORC1 pathway	[40]
	UCEC and the Human Protein Atlas database					
	Human endometrial adenocarcinoma cells (HEC-1A, Ishikawa)			Inhibits proliferation, migration and ROS production		
	HEC-1A/xenograft tumor model			suppresses tumor growth		
NSCLC	NSCLC patients:210	Down	Suppressor gene	Associates with tumor differentiation, advanced TNM stage, lymph node metastasis and overall survival; a favorable prognostic factor	N/A	[42]
Lung cancer	Lung cancer patients:77	Down:57 Up:20	suppressor gene	The low expression level of Sestrin2 associates with the poor survival	Suppresses Akt-mTOR-p70S6K signaling pathway	[43]
	Human lung adenocarcinoma cell (A549); human bronchial epithelial cell (BEAS-2B)			Suppresses the cell proliferation, migration, malignant transformation		
	BEAS-2B cell/xenograft tumor model			Suppresses the xenograft tumor growth in nude mice		
Lung cancer	Oncomine and the Human Protein Atlas database	Up	Oncogene	Overexpressed Sestrin2 has a poor prognostic value	N/A	[44]
	Human lung adenocarcinoma cell (A549)			Promotes cell proliferation, migration, sphere formation, and drug resistance		
Lung cancer	GEO (GSE 11969) and TCGA database	Up	Oncogene	The low expression of Sestrin2 showed longer survival	N/A	[45]
HCC	HCC patients:15	Down	suppressor gene	Associates with HBV/HCV infection, lymph node metastasis, tumor progression, and poor prognosis	N/A	[48]
HCC	HCC patients:14	Down	N/A	Participates in muscone-induced apoptosis and autophagy	Promotes cell apoptosis by PERK /ATF4/DDIT3 mediated ER stress; induces autophagy by Sestrin2/AMPK/mTORC1 pathway	[49]
HCC	HCC patients:30	Up	Oncogene	Correlates with proliferation marker Ki-67	Activates AKT and AMPK pathways	[31]
	HCC cell lines (Bel‐7404, HLF, HLE, SNU‐368, and Hep3B)			promotes cell proliferation and sorafenib resistance, inhibits apoptosis		

## The alteration of sestrin 2 gene expression in various cancers

Changes in sestrin 2 expression have been observed in numerous cancer tissues and cell lines and play a significant role in cell proliferation, invasion, metastasis, apoptosis, autophagy, anoikis resistance, drug resistance, oxidative stress, and endoplasmic reticulum (ER) stress. Sestrin 2 appears to be a novel prognostic marker for cancers (Table 1). In colorectal cancer (CRC) tissues and cell lines [30, 33], sebaceous gland carcinoma [34], neuroblastomas [35], bladder cancer tissues [36] and prostate cancer cells [37], sestrin 2 was downregulated in comparison with related non-cancerous tissues and cells. The lower expression of sestrin 2 was associated with advanced tumor stage, lymphatic and vascular invasion, liver metastasis, shorter disease-free and overall survival [30], upper eyelid involvement in sebaceous gland carcinoma [34], and radiosensitization [37]. Sestrin 2 was deemed a potential tumor suppressor that repressed cell proliferation, enhanced apoptosis, and induced autophagy [30, 34, 35]. Interestingly, multiple studies revealed higher expression levels of sestrin 2 in several cancers, including squamous cell carcinoma (SCC) and melanoma [38], metastatic melanoma tissues and cell lines [39], and endometrial cancer [40] compared with non-cancerous tissues or cell lines, and the increased expression of sestrin 2 was associated with poor prognosis [39, 40]. In these cancers, sestrin 2 was verified as an oncogene, which suppressed apoptosis and promoted drug [38] and anoikis resistance [39].

Notably, there have been conflicting reports regarding sestrin 2 alterations in lung and liver cancer. Primary lung cancer has the highest mortality worldwide, and non-small cell lung cancer (NSCLC) accounts for approximately 80% of cases [41]. Chen et al. found that the expression of sestrin 2 in NSCLC was markedly lower than that in corresponding non-cancerous lung tissues in 210 patients with NSCLC. Lower expression of sestrin 2 was correlated with poor differentiation, advanced TNM stage, and lymph node metastasis [42]. Lung cancer patients with a high expression of sestrin 2 had a longer overall survival rate than those with low expression of this protein [42, 43]. Interestingly, in contrast to this study, Chae et al. demonstrated that the expression of sestrin 2 in lung cancer was higher than in normal lung tissue by using the Oncomine and Human Protein Atlas databases. Furthermore, these authors showed that overexpressed sestrin 2 was a marker for poor prognosis in lung cancer by analyzing the PrognoScan database and Kaplan–Meier Plotter [44]. Additionally, by analyzing data from the Gene Expression Omnibus and Cancer Genome Atlas analysis, Lin et al. revealed that the survival time for lung cancer patients with lower sestrin 2 expression was significantly prolonged [45].

In 2018, liver cancer had the sixth highest incidence and the fourth highest mortality rate for cancers worldwide, and hepatocellular carcinoma (HCC) accounted for approximately 90% of cases [46, 47]. The expression of sestrin 2 in HCC was lower than that in non-cancerous tissues [48, 49], and lower sestrin 2 expression was associated with hepatitis B/hepatitis C viral infections, lymph node metastasis, tumor progression, and poor prognosis in HCC patients [48]. However, Dai et al. found that the levels of sestrin 2 were remarkably upregulated in HCC tissues and cell lines compared with the corresponding adjacent liver tissue and normal hepatocytes, and increased sestrin 2 expression was positively correlated with the proliferation marker Ki-67 [31].

## Inhibitory effects of sestrin 2 in cancer

### Sestrin 2 inhibits cell proliferation, migration, and invasion and induces apoptosis

The highly conserved serine-threonine kinase mTOR is frequently activated in cancer. Activated mTOR is involved in cell proliferation, migration and invasion, anti-apoptosis, and inhibition of autophagy [50, 51]. Two different protein complexes contain mTOR: mTORC1 and mTORC2 [52, 53]. The phosphoinositide 3-kinase (PI3K)/AKT and extracellular signal-regulated kinase/mitogen-activated protein kinase (ERK/MAPK) pathways activate mTORC1, whereas mTORC1 is inhibited by the AMPK pathway. Activated mTORC1 activates the downstream p70 ribosomal protein S6 kinase (p70S6K) and inhibits eucaryotic translation initiation factor 4E binding protein, which results in increased protein synthesis and tumor progression [54, 55]. Sestrin 2 was reported to participate in inhibiting tumor cell proliferation, migration, and invasion and inducing apoptosis. The anti-cancer functions of sestrin 2 are closely related to the inhibition of mTORC1 activity in endometrial cancer, CRC, and lung cancer.

Knockdown of sestrin 2 with short hairpin RNA (shRNA) induced migration and promoted proliferation by increasing mRNA expression of the proliferation marker Ki-67 and decreasing mRNA expression of the cyclin-dependent kinase inhibitors 1A and 1B (cell cycle-associated genes) in HEC-1A and Ishikawa endometrial cancer cell lines, which are dependent on the mTORC1 pathway [40]. Ro et al. revealed that sestrin 2 suppressed the development of colitis and CRC tumor growth by inhibiting ER stress and mTORC1, respectively [33]. Wei et al. found that sestrin 2 overexpression inhibited proliferation and activated apoptosis in CRC cells (SW620 and LoVo lines) by activating AMPK and inhibiting the mTORC1 pathway, which downregulated proliferating cell nuclear antigen and survivin proteins and upregulated caspase proteins 3, 7, and 9 [56]. Silencing sestrin 2 with shRNA facilitated proliferation and migration of lung epithelial cells. Moreover, this inhibitory role of sestrin 2 in lung epithelial cells was related to the regulation of the AKT-mTOR-p70S6K pathway [43]. In addition, sestrin 2 knockdown accelerated the xenograft tumor growth of HEC-1A [40], the CRC line SW620 [56], and bronchial epithelial line BEAS-2B [43] transplanted in Balb/c nude mice by targeting the mTORC1 mechanism. These studies indicated that sestrin 2 has potential anti-tumor effects in human endometrial cancer, CRC, and lung cancer cells by negatively regulating the mTORC1 pathway. Sestrin 2 may be a potential tumor suppressor in these types of cancer.

Interestingly, sestrin 2 also plays a significant role in growth inhibition of CRC, human head and neck cancer, medullary thyroid cancer, and breast cancer cells induced by the chemical drugs quercetin and 5-fluorouracil (5-FU), fisetin, 2-imino-6-methoxy-2H-chromene-3-carbothioamide (IMCA), and adiponectin, respectively. Furthermore, this growth inhibitory effect is closely related to the regulation of the AMPK/mTOR pathway. Quercetin is a flavonoid that exists in many fruits and vegetables and exerts anticancer effects [57]. Kim et al. demonstrated that quercetin suppressed proliferation and induced apoptosis in the CRC lines HCT116 and HT29 by targeting the sestrin 2/AMPK/mTOR pathway [58]. Seo and colleagues verified that 5-FU treatment significantly increased the expression of sestrin 2 in HCT116 and HT29 CRC cells by targeting p53, which consequently repressed CRC cell migration [59]. This latter study provides new evidence for the anti-tumor mechanism of 5-FU [59]. Fisetin, another key flavonoid, induced apoptosis in human head and neck cancer cells via upregulation of sestrin 2 expression and downregulation of phospho-mTOR and myeloid cell leukemia-1 protein [60]. IMCA inhibited proliferation and induced apoptosis by promoting the translocation of the orphan nuclear receptor 4A1 from the nucleus to the cytoplasm, further increasing sestrin 2 expression and phosphorylated-AMPK levels, and decreasing p70S6K in the TT thyroid cancer cell line [61].

Adiponectin, a cytokine primarily secreted by adipocytes, is well correlated with the occurrence and development of malignant tumors [62, 63]. Globular adiponectin suppressed the growth of breast cancer cells by inhibiting the activation of inflammasomes [64]. Adiponectin was reported to bind to receptors on the cell membrane, activate the downstream sestrin 2/AMPK pathway, inhibit ER stress, suppress inflammatory activity, inhibit proliferation, and promote apoptosis in breast cancer cell lines MCF-7, MDA-MB-231, and T47D [64].

Sestrin 2 has also been implicated in cold atmospheric plasma (CAP)-induced apoptosis of melanoma cells by activating the sestrin 2/nitric oxide synthase (iNOS) pathway [65]. Using CAP to treat cancer is a promising new technology in plasma medicine [66]. Xia et al. found that CAP induced caspase 3/8-mediated apoptosis in A375 and A875 melanoma cell lines by increasing sestrin 2 expression, which activated p38 MAPK signaling and increased the expression of downstream Fas, Fas ligand, and iNOS; this process was reversed by using short interfering RNA targeting sestrin 2 [65].

Sestrin 2 expression was upregulated by the different treatment factors described above, but the mechanism of upregulation was not consistent. Upregulation of sestrin 2 protein by quercetin was related to the increase in reactive oxygen species (ROS) production [58]. Sestrin 2 mRNA upregulation by ICMA and increased mRNA and protein levels by 5-FU were dependent on the tumor-suppressor protein p53 [59, 61]. The specific mechanisms remain unclear for upregulation of sestrin 2 protein by globular adiponectin [64] and increased sestrin 2 mRNA and protein levels by fisetin and CAP [60, 65]. In conclusion, the upregulation of sestrin 2 expression is an essential mediator for inhibiting cancer cell growth through the AMPK/mTOR or iNOS pathway and may relate to ROS induction and p53 activation.

In addition to regulating mTOR and iNOS pathways, sestrin 2 has been shown to promote cancer cell apoptosis by targeting X-linked inhibitor of apoptosis protein (XIAP) and suppress cell migration and invasion by targeting hypoxia inducible factor-1α (HIF-1α). XIAP is a powerful cellular inhibitor of apoptosis that suppresses caspase 3, 7, and 9 activity [67]. Ding et al. reported that sestrin 2 stimulated the degradation of XIAP through the lysosomal pathway and promoted death receptor-induced apoptosis of lung adenocarcinoma cell lines H460 and A549 [68]. In hypoxic microenvironments, tumor cells overproduce HIF-1α, which is conducive to survival [69]. Decreasing HIF-1α expression is an efficient strategy for inhibiting tumor cell survival and invasion [70]. Seo et al. revealed that sestrin 2 suppressed HCT116 and HT29 CRC cell migration and invasion in vitro and inhibited tumor growth in vivo by promoting the degradation of HIF-1α through the AMPK-prolyl hydroxylase pathway [71].

**Fig. 2:**
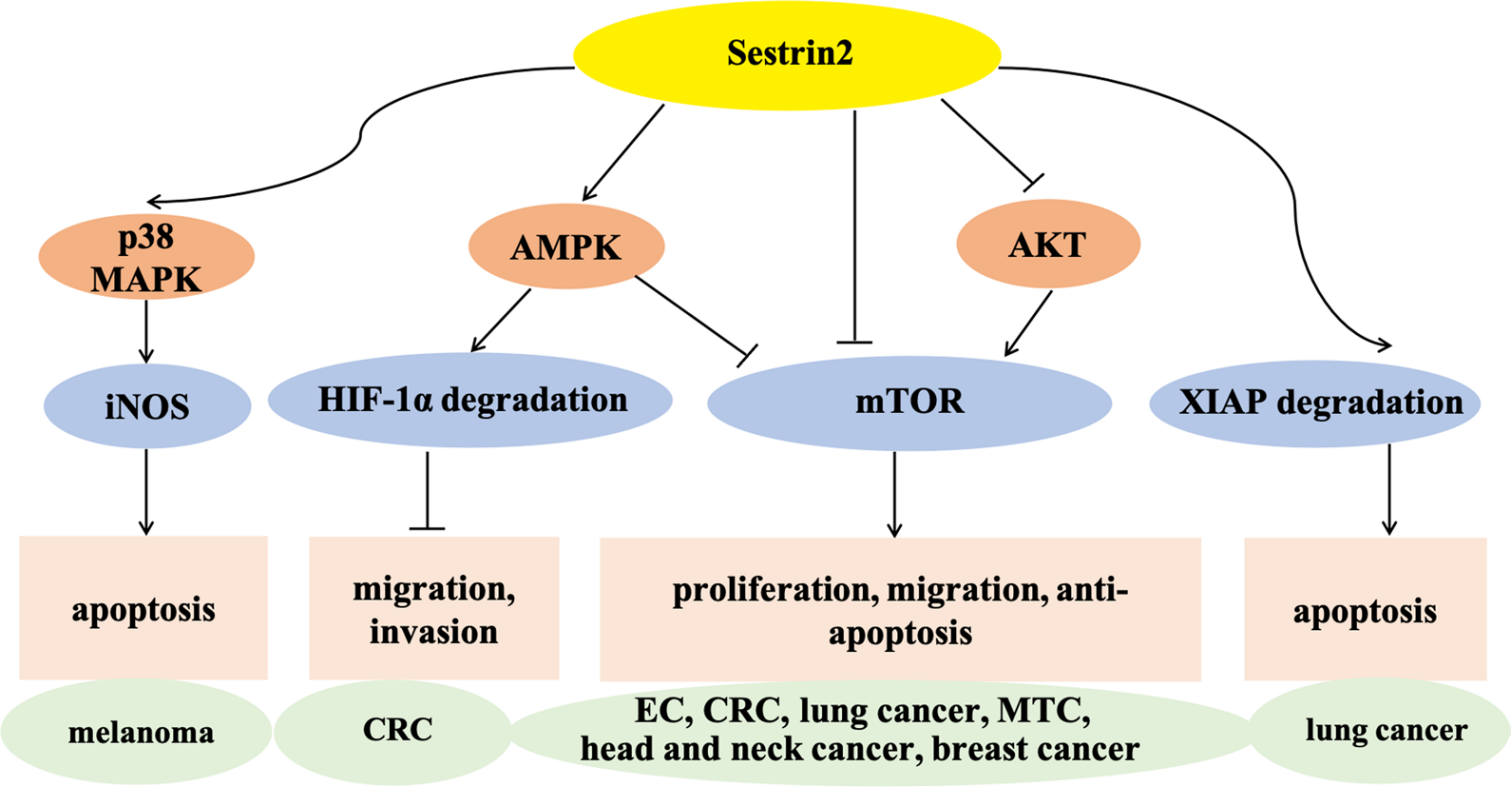
Pathways of sestrin 2-mediated suppression of different cancers by inhibiting cell proliferation, migration, and invasion and inducing apoptosis

Overall, sestrin 2 plays a crucial role in suppressing tumor growth by inhibiting cellular proliferation, migration, and invasion, and inducing apoptosis. The signaling pathways involved in sestrin 2-mediated suppression of different cancers are summarized in Fig. 2.

### Sestrin 2 participates in autophagy induction

Autophagy, or autophagocytosis, is another type of programmed cellular death, which is different from apoptosis. Autophagy maintains homeostasis by using lysosomes to degrade misfolded proteins and damaged organelles in the cytoplasm. Sestrin 2 is critical for the regulation of autophagy [72, 73]. Zhang et al. reported that c-Jun N-terminal kinase mediated the autophagy induced by excisanin A and serum deprivation through a sestrin 2-dependent mechanism in the CNE1 and CNE2 human nasopharyngeal carcinoma cell lines [29]. Additionally, the inhibition of lysine-specific demethylase 1 promoted autophagy through the sestrin 2-mTORC1 signaling pathway in the neuroblastoma cell lines SH-SY5Y, SHEP Tet-21/N, and SK-N-BE [35].

**Fig. 3:**
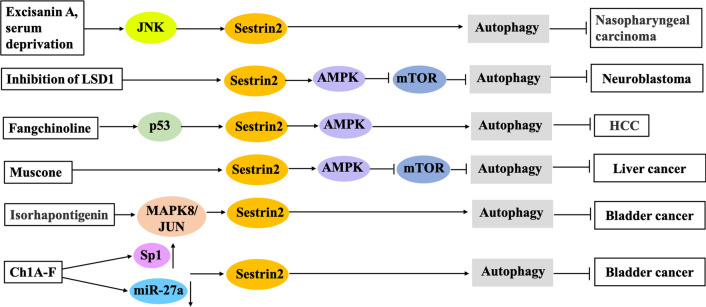
Pathways involved in sestrin 2-mediated promotion of autophagy in different cancers

Sestrin 2-mediated autophagy is associated with antitumor activities of drugs [14]. Fangchinoline, a bisbenzylisoquinolin alkaloid from the dried roots of *Stephania tetrandra* S. Moore, has anti-inflammatory, antihyperglycemic, antioxidant, and anticancer activities [74–76]. Wang et al. elucidated a novel mechanism of action for fangchinoline-induced autophagy in HepG2 and PLC/PRF/5 HCC cells that used p53/sestrin 2/AMPK signaling [76]. Muscone, the main active ingredient in musk, promoted apoptosis of HepG2 hepatoma cells through the phosphorylated-protein kinase RNA-like endoplasmic reticulum kinase/activating transcription factor 4/DNA damage inducible transcript 3 mediated ER stress pathway and induced autophagy through the sestrin 2/AMPK/mTORC1 signaling pathway [49]. Apoptosis induced by muscone may be partially dependent on autophagy [49]. In vivo experiments with nude mice confirmed that muscone suppressed the growth of transplanted tumors. Furthermore, sestrin 2 was identified as a potential candidate gene for the diagnosis and therapy of liver cancer [49]. Isorhapontigenin (ISO), found in Chinese herbs, has anti-bladder cancer properties. After treatment of UMUC3 and T24T bladder cancer cells with ISO, sestrin 2 expression was elevated by activation of the MAPK8/JUN pathway, and activated sestrin 2 induced autophagy and inhibited the growth of bladder cancer cells [36]. Additionally, a new antitumor compound, known as ChlA-F, increased sestrin 2 transcription by activating transcription factor specificity protein 1. Ch1A-F enhanced sestrin 2 stability by inhibiting microRNA (miR)-27a-mediated degradation of sestrin 2 mRNA, which resulted in the increased expression of sestrin 2 protein, activation of the autophagy pathway, and inhibition of anchorage-independent growth of bladder cancer cell lines, including RT4, T24T, and UMUC3 cells [77]. The pathways of sestrin 2-mediated promotion of cell autophagy are summarized in Fig. 3.

### Sestrin 2 enhances radiosensitivity

Radiation therapy is one of the critical methods for comprehensive treatment of cancer. It can improve a cancer patient’s quality of life and long-term survival rate by causing DNA damage, depriving cells of their reproductive capacity, and, ultimately, killing the tumor cells. Because of the different radiosensitivity between individuals and/or tumor cells, some patients will respond poorly to radiation therapy. Therefore, it is imperative to explore influential factors and mechanisms to reduce tumor radioprotection and enhance tumor radiosensitivity [37, 78]. One study showed that sestrin 2 increased the radiosensitivity of MCF-7 breast cancer cells. Sestrin 2 was implicated in radiation-induced death of breast cancer cells by stabilizing the AMPK complex and/or enhancing AMPK expression. Sestrin 2 associated with AMPK to inhibit mTOR signaling and enhanced radiation therapy-induced tumor cell death [78]. Another study found that sestrin 2 expression was low in prostate cancer cells lines, including PC3, LNCaP clone FGC, and DU145. Overexpression of sestrin 2 reduced the proliferation of PC3 cells and increased radiosensitivity [37].

## Promotive effects of sestrin 2 in cancer

### Sestrin 2 promotes tumor growth

Interestingly, the role and mechanisms of action of sestrin 2 in mediating cell growth and survival are still controversial. Some studies identified sestrin 2 as an oncogene, which caused accelerated tumor cell growth and migration and suppressed apoptosis. The tumor suppressor gene phosphatase and tensin homolog deleted on chromosome ten (PTEN) negatively regulates the PI3K/AKT pathway and participates in cell proliferation, survival, migration, and metabolism [79]. Zhao et al. reported that sestrin 2 acted as an oncogene in SCC and melanoma. Sestrin 2 knockdown facilitated apoptosis induced by vemurafenib and 5-FU in human melanoma cells (A375 and MEL624 lines) and the SCC cell line A431 [38], respectively, and suppressed the growth of A431 and A375 cells xenografted in nude mice [38]. Sestrin 2 activated AKT by decreasing PTEN membrane recruitment [38]. Therefore, the positive effect of sestrin 2 on AKT activity may be related to survival of SCC and melanoma. Using the NK-92 cell line, Wang et al. described the role of sestrin 2 in natural killer cell activity against ovarian cancer. Sestrin 2 promoted ovarian cancer cell survival by suppressing the anti-tumor effects of NK-92 cells through activation of AMPK and inhibition of the mTORC1 pathway [80]. Sorafenib is an effective drug that inhibits tumor growth and angiogenesis of HCC [47, 81]. Sorafenib treatment increased the expression of sestrin 2 in human HCC lines Bel-7404 and SNU-368, and knockdown of sestrin 2 enhanced growth inhibition and apoptosis induced by sorafenib. Therefore, sestrin 2 may have potential tumorigenic effects in HCC [31]. Sestrin 2 also has a potentially oncogenic function in lung cancer. Knockdown of sestrin 2 with shRNA inhibited proliferation, migration, and sphere formation in A549 cells [44]. In pancreatic cancer, overexpression of sestrin 2 promoted PANC-1 cells proliferation and increased glycolysis, and mTOR inhibitors suppressed these effects in vitro. Additionally, the knockdown of sestrin 2 inhibited the growth of pancreatic cancer in vivo. This latter study indicated that the mTOR signaling pathway participated in sestrin 2-mediated promotion and development of pancreatic cancer [82].

### Sestrin 2 promotes anoikis and drug resistance

Metastasis is an important feature of malignant tumors and indicates a poor prognosis. Anoikis resistance is one of the pivotal mechanisms for cancer metastasis [83]. Sestrin 2 facilitates metastasis and cancer anoikis resistance. It was shown that the interaction between sestrin 2 and miR-141 affected the anoikis resistance of human endometrial cancer cell lines KLE, RL-95-2, Ishikawa, and AN3CA; inhibiting miR-141 increased sestrin 2 protein expression, resulting in enhanced anoikis resistance [32]. Another study demonstrated that sestrin 2 promoted anoikis resistance in melanoma cells and contributed to melanoma metastasis in vivo [39]. The detachment of metastatic melanoma cells from the extracellular matrix can induce the up-regulation of sestrin 2 expression because of suspension stress. The specific molecular mechanisms by which sestrin 2 inhibits anoikis include the reduction of intracellular ROS levels and regulation of endogenous apoptosis-related proteins, including B-cell lymphoma 2 and B-cell lymphoma 2 associated X-protein [39].

**Fig. 4:**
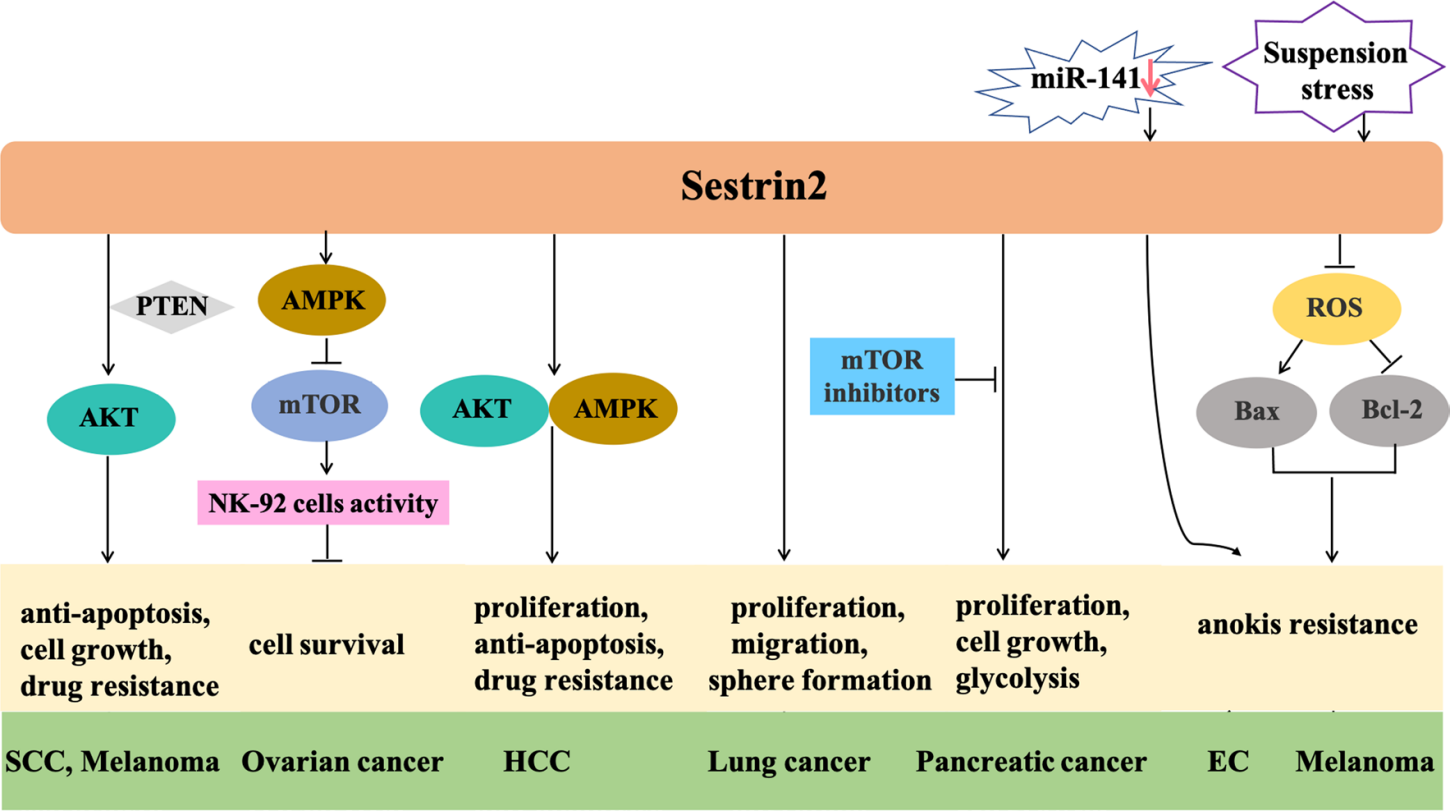
Pathways of sestrin 2 in different cancers that promote anoikis resistance, cell growth, and drug resistance

Drug resistance is an important reason for therapeutic failure in cancer chemotherapy [84, 85]. Exploring the reasons behind drug resistance is of great significance to fundamentally solve this problem and improve the survival rate of patients. Sestrin 2 is a crucial target molecule for chemotherapeutic drug resistance because it activates the AKT and/or AMPK pathways [31, 38]. In human SCC and melanoma cells, sestrin 2 induced chemotherapeutic drug resistance by activating the AKT pathway and regulating PTEN activity [38]. In HCC, sestrin 2 was involved in primary resistance to sorafenib by activating the AKT and AMPK pathways [31]. In A549 lung cancer cells, sestrin 2 knockdown decreased the mRNA expression of ATP-binding cassette transporter ABCG and ABCA2 (drug resistance marker genes) and increased sensitivity to doxorubicin [44]. Taken together, targeting the sestrin 2 gene may be a valuable method to overcome primary resistance to chemotherapy drugs. Figure 4 shows the pathways of sestrin 2-mediated tumorigenesis that promote tumor growth, anoikis resistance, and drug resistance.

### Sestrin 2 is required for cancer cell survival under stress conditions

Sestrin 2 is a stress-induced protein family member activated by diverse stressors, such as glucose starvation, nutritional deficiency, ER stress, oxidative stress, hypoxia, and DNA damage [14, 18]. Activated sestrin 2 has been found to be beneficial for tumor cell survival under diverse stress conditions [86–89].

Glucose is one of the primary sources of energy for cancer cell growth. Ben-Sahra et al. demonstrated that sestrin 2 was a key molecular for cancer cell survival when glycolysis was blocked [86]. The expression of sestrin 2 was upregulated by an AKT-dependent but p53-independent mechanism, and sestrin 2 was essential for mTOR suppression under a state of energy stress caused by glycolysis inhibition [86]. Currently, sestrin 2 is regarded as a novel energy stress sensor [86]. Recently, a study by Kumar et al. showed that under glucose starvation conditions (with adequate glutamine), sestrin 2 promoted HepG2 cell survival by activating PPAR-γ coactivator-1 alpha (a stress sensor in cancer cells) through the modulation of glutamine metabolism [89]. This study demonstrated the crucial role of sestrin 2 in regulating cancer cell survival under glucose starvation conditions.

Glutamine is an essential nutrient for tumor cell proliferation. However, how tumor cells survive during glutamine deficiency remains unknown. Byun et al. revealed that after glutamine deficiency, sestrin 2 expression was upregulated through a ROS-p38 MAPK-CCAAT/enhancer-binding protein β-dependent pathway. The depletion of glutamine resulted in the binding of sestrin 2 to mTORC2 to increase the stability of the sestrin 2 protein and reduce the activity of mTORC1, thus, preventing lung cancer cell death [88]. The differential regulation of mTORC1 and mTORC2 by sestrin 2 can prevent ATP depletion and maintain redox balance [88]. The positive feedback loop between sestrin 2 and mTORC2 is vital for lung cancer cell survival during glutamine deficiency [88].

Kim et al. explored the response of cancer cells to heme iron-induced stress. Hemin (Fe^3+^ heme) induced the expression of sestrin 2 by activating ROS and nuclear factor (erythroid-derived 2)-like 2, and, together with hemin, sestrin 2 overexpression protected colon cancer cells from death including HCT116 and RKO cells and promoted MC38 tumor growth both in vitro and in vivo. This study suggested that sestrin 2 has a promotive effect on colon cancer during high iron conditions [28].

ER stress is involved in various biological processes of cancer and is closely related to biological functions, such as proliferation, apoptosis, drug resistance, and autophagy [90–92]. Sestrin 2 expression is upregulated under ER stress in cancer cells through the regulation of the activating transcription factor 4 [93], inositol-requiring enzyme 1/X-box binding protein 1, and protein kinase RNA-like endoplasmic reticulum kinase signaling pathways [87, 94]. Sestrin 2 knockdown reduced ER stress-induced autophagy and promoted ER stress-induced cell death by activating the mTORC1 pathway in breast cancer cells lines HCC1806 and MCF7 [87]. This study emphasizes that sestrin 2 induction mediated by ER stress contributes to tumor cell survival and that targeting sestrin 2 may be a mechanism for enhancing ER stress-induced cell death in breast cancer cells [87].

**Fig. 5:**
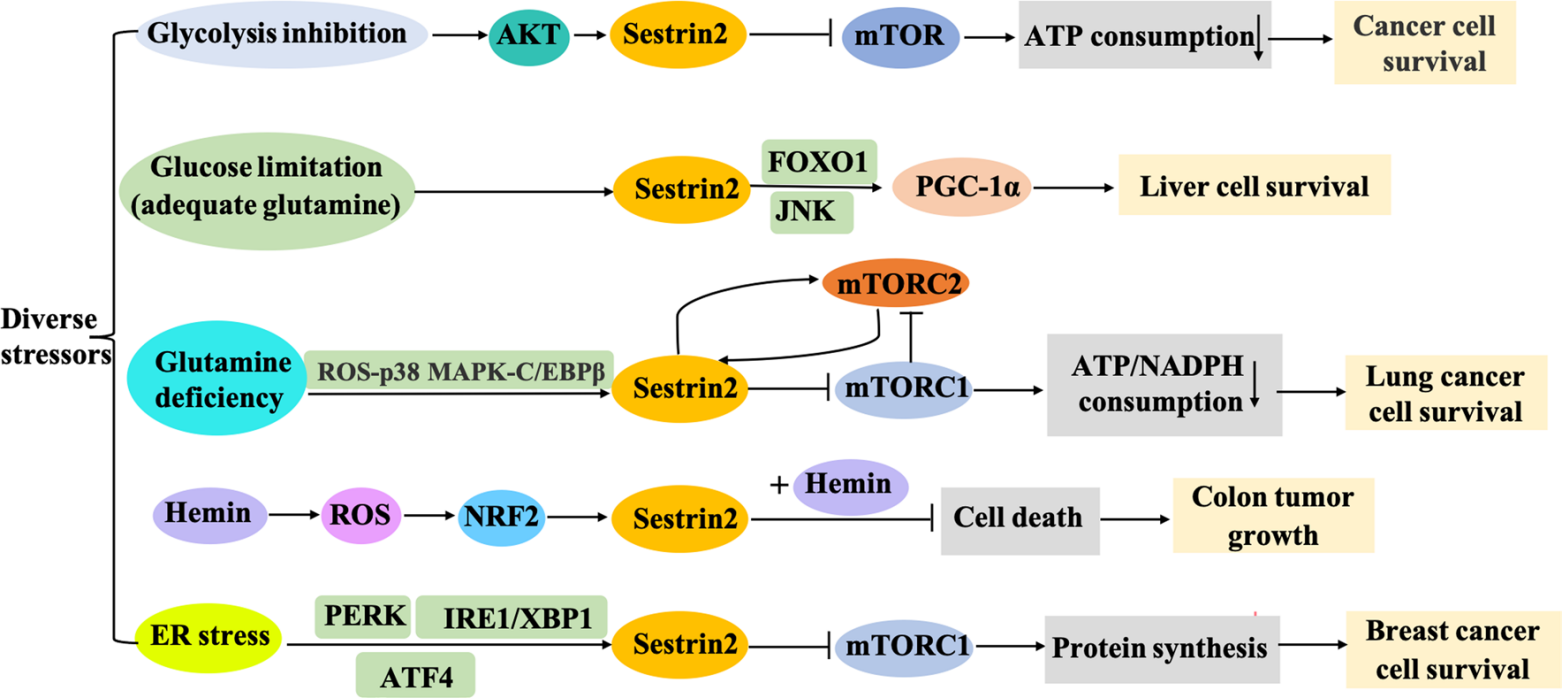
Sestrin 2 pathways that mediate cancer cell survival under stress conditions

Together, these studies revealed that sestrin 2 is a vital regulator of cancer cell survival under the conditions of glycolysis inhibition, glutamine deficiency, glucose starvation, high iron, and ER stress. These pathways are summarized in Fig. 5.

## Conclusion

In this review, we summarized the dual roles and mechanisms of sestrin 2 in various cancers. There are currently many unanswered questions regarding the role of sestrin 2 in tumors. First, it will be necessary to understand why the expression of sestrin 2 is inconsistent in different tumors. The evaluation of a greater number of clinical samples will be required in subsequent studies. Second, the functions of sestrin 2 in different tumors requires further exploration. Some studies have shown that sestrin 2 plays a role as a tumor suppressor gene in bladder and prostate cancer. However, sestrin 2 acts as an oncogene in SCC, pancreatic cancer, and ovarian cancer. It remains controversial whether sestrin 2 serves as a tumor-suppressing gene or oncogene in lung, liver, colorectal, breast, melanoma, and endometrial cancers. Furthermore, it is unclear whether sestrin 2 acts as a tumor suppressor or promoter in thyroid cancer, head and neck cancer, neuroblastoma, and nasopharyngeal carcinoma, and verification requires more data. Third, the mechanisms by which sestrin 2 functions should be clarified. According to this literature review, the mechanisms used by sestrin 2 to inhibit tumor growth are mainly associated with mTOR, iNOS, XIAP, and HIF-1α signaling pathways. However, the mechanisms by which sestrin 2 promotes tumor growth are relatively complex, especially under stress conditions. When tumors are under stress, such as during hypoxia or nutritional deficiency, they can cope with the crisis through a series of protective mechanisms, including reducing metabolic energy consumption, delaying cell growth, and inhibiting apoptosis. Sestrin 2 can be regarded as a protective factor for cell survival during stressful conditions. We discussed the possible mechanisms of sestrin 2 function and considered that differences in metabolic status and tumor types may be the reasons for the contradictory roles of sestrin 2. However, to a certain extent, this conclusion is not satisfactory because of the lack of more favorable experimental evidence and the role of sestrin 2 in cancer requires further validation.

Sestrin 2 plays an essential role in various tumors and is a promising diagnostic and therapeutic target. For diagnosis, sestrin 2 protein may be useful as an auxiliary characteristic to determine tumor classification and prognosis. For treatment, sestrin 2 can be used as an effective target for the development of anticancer drugs. However, the function of sestrin 2 in regulating cancer is bidirectional, and the underlying mechanisms need further exploration and verification.

## Data Availability

Not applicable.

## Abbreviations

mTOR: Mammalian target of rapamycin
mTORC1: Mammalian target of rapamycin complex 1
AMPK: 5ʹ-Adenosine monophosphate-activated protein kinase
TNM: Tumor, node, and metastasis
ER: Endoplasmic reticulum
CRC: Colorectal cancer
SCC: Squamous cell carcinoma
NSCLC: Non-small cell lung cancer
HCC: Hepatocellular carcinoma
PI3K: Phosphoinositide 3-kinase
ERK: Extracellular signal-regulated kinase
MAPK: Mitogen-activated protein kinase
p70S6K: P70 ribosomal protein S6 kinase
shRNA: Short hairpin RNA
5-FU: 5-Fluorouracil
IMCA: 2-Imino-6-methoxy-2H-chromene-3-carbothioamide
CAP: Cold atmospheric plasma
iNOS: Nitric oxide synthase
ROS: Reactive oxygen species
XIAP: X-linked inhibitor of apoptosis protein
HIF-1α: Hypoxia inducible factor-1α
ISO: Isorhapontigenin
miR: MicroRNA
PTEN: Tensin homolog deleted on chromosome ten
CRC: Colorectal cancer
EC: Endometrial cancer
MTC: Medullary thyroid cancer
EC: Endometrial cancer
FOXO1: Forkhead box 1 transcription factor
C/EBPβ: CCAAT/enhancer binding protein β
PERK: Phosphorylated-protein kinase RNA-like endoplasmic reticulum kinase
IRE1: Inositol-requiring enzyme 1
XBP1: X-box binding protein 1
ATF4: Activating transcription factor 4
Up: Upregulation of Sestrin2
Down: Downregulation of Sestrin2
SGC: Sebaceous gland carcinoma
NB: Neuroblastomas
SCC: Skin squamous cell carcinoma
LSD1: Lysine-specific demethylase
TCGA: The Cancer Genome Atlas
UCEC: TCGA-Uterine Corpus Endometrial Carcinoma
GEO: Gene Expression Omnibus
GSE: GEO Series
HBV: Hepatitis B viral
HCV: Hepatitis C viral
DDIT3: DNA damage inducible transcript 3
N/A: not available
Nrf2: Nuclear factor E2-related factor 2
AP-1: Activating protein-1
Sp-1: Specificity protein 1
